# P-1321. The Infectious Disease Needs of Adult Ukrainian Refugees in a Tertiary Centre in the West of Ireland

**DOI:** 10.1093/ofid/ofae631.1500

**Published:** 2025-01-29

**Authors:** Siobhan Quirke, Lisa Duffy, Ellen Walsh, Mary Bohan Keane, Margaret Scarry, Nicola Boyle, Catherine Langan, Katie McDonough, Helen Tuite, David Gallagher, Geraldine Moloney, John Lee, Catherine Fleming

**Affiliations:** HSE, Dublin, Dublin, Ireland; HSE, Dublin, Dublin, Ireland; HSE, Dublin, Dublin, Ireland; HSE, Dublin, Dublin, Ireland; HSE, Dublin, Dublin, Ireland; HSE, Dublin, Dublin, Ireland; HSE, Dublin, Dublin, Ireland; HSE, Dublin, Dublin, Ireland; HSE, Dublin, Dublin, Ireland; HSE, Dublin, Dublin, Ireland; HSE, Dublin, Dublin, Ireland; HSE, Dublin, Dublin, Ireland; Galway University Hospital, Galway, Galway, Ireland

## Abstract

**Background:**

Since February 2022 6.5 million have fled Ukraine; 104’ 870 to Ireland (2% of Irish population) [1, 2] The surge in those seeking temporary protection, coupled with infectious disease epidemiology in Ukraine; high prevalence of blood borne viruses and TB has placed strain on host countries' health services. [3, 4] 18 ‘840 (18% of National total, 0.37% of Irish population) are accommodated within our catchment area. The objective of this study was to evaluate case complexity, barriers to access and impact on service by this population.

HIV, HCV and HBV prevalence, control and management

**Figure:**
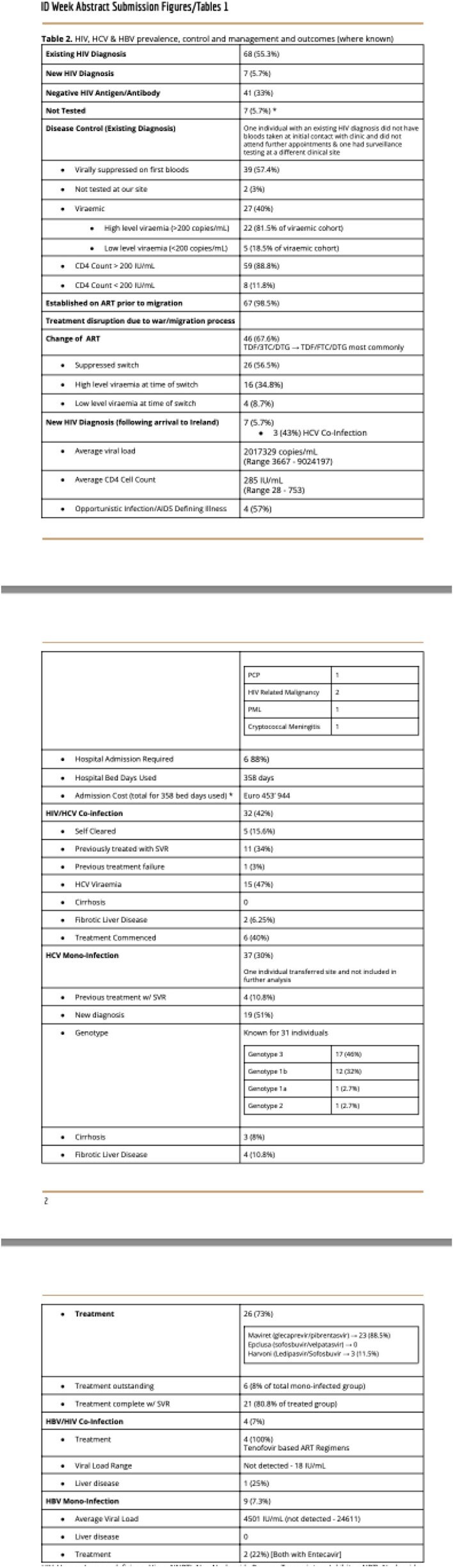

**Methods:**

We performed a retrospective chart review using our electronic patient record, compiled data on a protected spreadsheet and results were collated.

Tuberculosis prevalence, susceptibility, site and management

**Figure:**
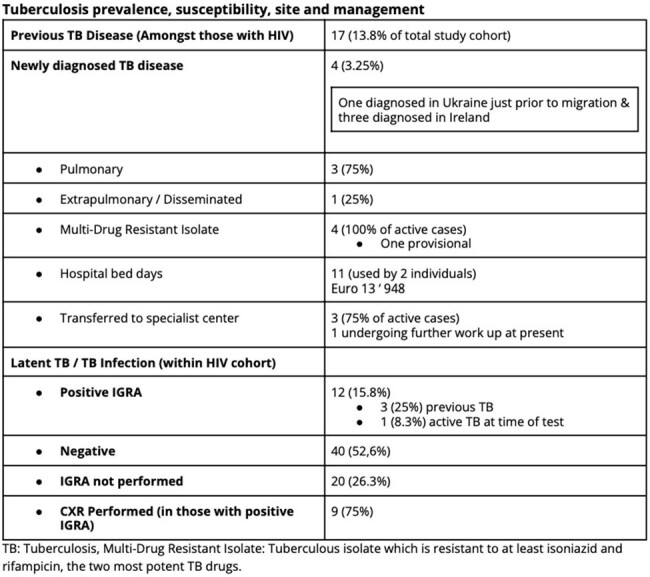

**Results:**

123 adult Ukrainian refugees have newly attended our service since February 2022 - April 2024, 2.03 new patients weekly; 63/123 (51.2%) are female. The mean age is 44.7 years (18 - 79). 76 (61.8%) have HIV (PLWH); 68 (89.5%) known diagnoses; 39 (57.3%) of whom were virally suppressed on ART and 27 (40%) were viraemic as a result of treatment interruption, 2 (2.9%) had no blood testing with us. 7 (5.7%) were newly diagnosed following arrival to Ireland; 4 (57%) with advanced disease (mean CD4 Count 285 IU/mL [28 - 753]). Thirty two (42%) PLWH are co-infected with HCV, 4 (5.3%) with HBV. 37 (30%) attended with HCV mono-infection, 9 (7.3%) with HBV mono-infection. 17 PLWH (22.4%) report previously treated TB. We have seen 4 (3.25%) cases of active TB; three of MDR pulmonary disease and results are pending for the fourth case. 4, 10.5% of women living with HIV in this cohort have had pregnancies; 2 (5.3% of WLH) are currently pregnant (both live in Donegal; average 165.6 miles from clinic). The average distance traveled to our clinic is 64.6 miles (Range 0.62 - 189); based on current geographic distribution of the group 50.4% (62/123) can access public transport that would allow them to reach our clinic within 2 hours. There was no significant association between access to public transport and missed clinic appointments (p = 0.64). 115/123 (93.5%) have a need for an interpreter. Since February 2022 26 (21%) have required hospital admission with an average length of stay of 22.9 days and a total of 597 bed days used.

**Figure:**
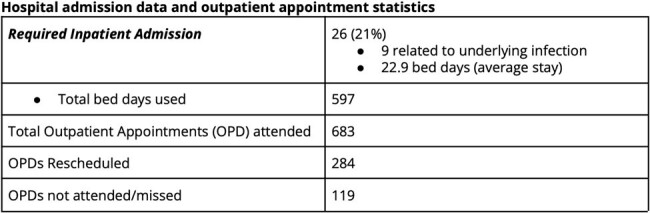

**Conclusion:**

We have identified significant case complexity and ongoing high ID service need for this cohort.

Cohort Demographics

**Figure:**
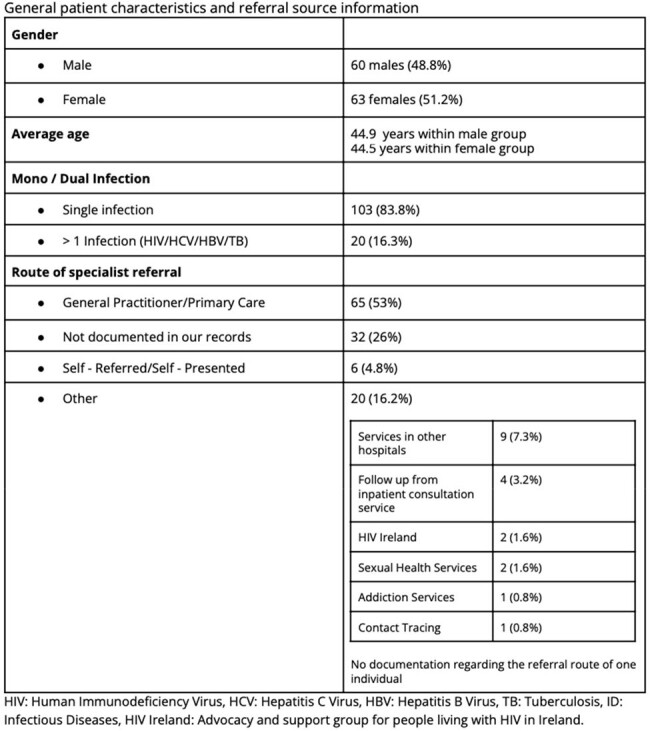

**Disclosures:**

**All Authors**: No reported disclosures

